# Spatiotemporal Distribution of Pseudomonas aeruginosa Alkyl Quinolones under Metabolic and Competitive Stress

**DOI:** 10.1128/mSphere.00426-20

**Published:** 2020-07-22

**Authors:** Tianyuan Cao, Jonathan V. Sweedler, Paul W. Bohn, Joshua D. Shrout

**Affiliations:** a Department of Chemistry and Biochemistry, University of Notre Dame, Notre Dame, Indiana, USA; b Department of Chemistry, University of Illinois at Urbana-Champaign, Urbana, Illinois, USA; c Beckman Institute for Advanced Science and Technology, University of Illinois at Urbana-Champaign, Urbana, Illinois, USA; d Department of Chemical and Biomolecular Engineering, University of Notre Dame, Notre Dame, Indiana, USA; e Advanced Diagnostics and Therapeutics, University of Notre Dame, Notre Dame, Indiana, USA; f Department of Civil and Environmental Engineering and Earth Sciences, University of Notre Dame, Notre Dame, Indiana, USA; g Department of Biological Sciences, University of Notre Dame, Notre Dame, Indiana, USA; h Eck Institute for Global Health, University of Notre Dame, Notre Dame, Indiana, USA; University of Iowa

**Keywords:** PQS, HQNO, *Staphylococcus aureus*, polymicrobial, quorum sensing, Raman spectroscopy, principal-component analysis, chemical imaging

## Abstract

Pseudomonas aeruginosa is an opportunistic human pathogen important to diseases such as cystic fibrosis. P. aeruginosa has multiple quorum-sensing (QS) systems, one of which utilizes the signaling molecule 2-heptyl-3-hydroxy-4-quinolone (*Pseudomonas* quinolone signal [PQS]). Here, we use hyperspectral Raman imaging to elucidate the spatiotemporal PQS distributions that determine how P. aeruginosa regulates surface colonization and its response to both metabolic stress and competition from other bacterial strains. These chemical imaging experiments illustrate the strong link between environmental challenges, such as metabolic stress caused by nutritional limitations or the presence of another bacterial species, and PQS signaling. Metabolic stress elicits a complex response in which limited nutrients induce the bacteria to produce PQS earlier, but the bacteria may also pause PQS production entirely if the nutrient concentration is too low. Separately, coculturing P. aeruginosa in the proximity of another bacterial species, or its culture supernatant, results in earlier production of PQS. However, these differences in PQS appearance are not observed for all alkyl quinolones (AQs) measured; the spatiotemporal response of 2-heptyl-4-hydroxyquinoline *N*-oxide (HQNO) is highly uniform for most conditions. These insights on the spatiotemporal distributions of quinolones provide additional perspective on the behavior of P. aeruginosa in response to different environmental cues.

**IMPORTANCE** Alkyl quinolones (AQs), including *Pseudomonas* quinolone signal (PQS), made by the opportunistic pathogen Pseudomonas aeruginosa have been associated with both population density and stress. The regulation of AQ production is known to be complex, and the stimuli that modulate AQ responses are not fully clear. Here, we have used hyperspectral Raman chemical imaging to examine the temporal and spatial profiles of AQs exhibited by P. aeruginosa under several potentially stressful conditions. We found that metabolic stress, effected by carbon limitation, or competition stress, effected by proximity to other species, resulted in accelerated PQS production. This competition effect did not require cell-to-cell interaction, as evidenced by the fact that the addition of supernatants from either Escherichia coli or Staphylococcus aureus led to early appearance of PQS. Lastly, the fact that these modulations were observed for PQS but not for all AQs suggests a high level of complexity in AQ regulation that remains to be discerned.

## Abstract (precis)

Alkyl quinolones (AQs), including *Pseudomonas* quinolone signal (PQS), made by the opportunistic pathogen Pseudomonas aeruginosa have been associated with both population density and stress. The regulation of AQ production is known to be complex, and the stimuli that modulate AQ responses are not fully clear. Here, we have used hyperspectral Raman chemical imaging to examine the temporal and spatial profiles of AQs exhibited by P. aeruginosa under several potentially stressful conditions. We found that metabolic stress, effected by carbon limitation, or competition stress, effected by proximity to other species, resulted in accelerated PQS production. This competition effect did not require cell-to-cell interaction, as evidenced by the fact that the addition of supernatants from either Escherichia coli or Staphylococcus aureus led to early appearance of PQS. Lastly, the fact that these modulations were observed for PQS but not for all AQs suggests a high level of complexity in AQ regulation that remains to be discerned.

## INTRODUCTION

Pseudomonas aeruginosa is a ubiquitous Gram-negative bacterium and an opportunistic human pathogen that can be found in soil and freshwater and also in infection environments, such as the lungs of cystic fibrosis patients (1, 2). Like many other bacterial species, P. aeruginosa can coordinate group behaviors, such as surface movement, biofilm formation, and virulence factor production, using several mechanisms. One mechanism of coordination utilizes a communication system known as quorum sensing (QS), whereby individual cells, secrete, release, and sense chemical signal molecules (3, 4), enabling responses to environmental challenges in a cooperative and coordinated way to improve bacterial survival (5–8). P. aeruginosa uses four interconnected QS signaling systems, i.e., the Las, Rhl, PQS (*Pseudomonas* quinolone signal), and IQS (integrated quorum-sensing) systems (9), which are organized in a multilayered and intertwined hierarchy.

The PQS system uses 2-alkyl-4(1*H*)-quinolones (AQs) (10) to mediate bacterial behaviors, including iron chelation, cytotoxicity, and other functions associated with virulence (11–13). The regulation of AQ production, not just PQS production, is complex. Synthesis of the AQs first requires activity encoded by the *pqsABCD* operon to act upon the precursor anthranilic acid (14, 15). Induction of *pqsABCD* is regulated by the Las quorum-sensing regulon via the transcriptional regulator PqsR (MvfR) (15, 16). Studies have shown that PqsR also plays a role in many biological activities involving another AQ, namely, 2-heptyl-4-hydroxyquinoline *N*-oxide (HQNO) (14, 17–19). While the production of both PQS and HQNO involves the transformation of modified anthranilate precursors by PqsABC, their synthesis pathways are known to diverge in one or more ways, since PQS and HQNO require the activities of PqsH and PqsL, respectively (14, 19).

Recent studies have indicated that PQS production also depends on the IQS signal (9). While phosphate limitation induces PQS production (20), initiation of the stringent response by starvation leads to the repression of AQ production (21). Thus, while some “insulated” actions of PQS regulation are clear (22), there is still much to learn about the factors and circumstances that determine how P. aeruginosa activates the PQS pathway in response to environmental challenges (23).

Under conditions that promote the collective movement described as swarming, PQS has been shown to promote a protective response to some antibiotic classes (but not all) (23, 24) and also to protect against phage infection (24). These findings are also related to earlier work reporting that PQS generally limits swarming expansion (18). Additionally, previous studies from our laboratory (12, 25) have produced strong evidence that P. aeruginosa secretes a characteristic sequence of AQs in the first 96 h in monocultures grown on surfaces. However, in most growth environments, P. aeruginosa is likely to coexist and compete with other bacterial species. Detailed examinations of some cocultures and mixed cultures of bacteria have suggested that bacterial species can alter their QS system responses under environmentally competitive conditions in order to respond to messages from other bacterial species, altering their behavior accordingly (5, 26, 27).

Our laboratories have established the utility of combined multimodal chemical imaging as a tool for discerning the spatial and temporal distributions of a range of bacterial compounds. In the present work, we apply comprehensive hyperspectral Raman imaging to characterize the spatiotemporal distribution of AQs produced in response to different stresses. Specifically, we examined the behavior of P. aeruginosa under two kinds of environmental challenges: metabolic stress induced by nutrient limitations and the competitive stress induced by coculturing P. aeruginosa with Escherichia coli. The spatiotemporal chemical information we have obtained about secreted AQs enables us to conclude that while nutrient limitation represses all AQ production during surface growth, other environmental challenges do not modulate PQS and HQNO responses equally.

## RESULTS AND DISCUSSION

### Surface growth in the presence of E. coli elicits earlier production of PQS in P. aeruginosa.

While many reports have annotated PQS as a stress response (16, 24, 28), the triggers for AQ production and PQS response are not clear. Here, we analyzed the spatiotemporal aspects of two AQs: 2-heptyl-3-hydroxy-4(1*H*) (PQS) and 2-heptyl-4-hydroxyquinoline *N*-oxide (HQNO). We examined the PQS and HQNO responses of P. aeruginosa growing in proximity to E. coli K-12, a model lab bacterium that we used as a nonspecific competitor of P. aeruginosa. These species were cultured simultaneously by inoculation at a distance of 12 mm from each other on a semisolid agar medium (see Fig. S1 in the supplemental material). At time points (*t*) of 24 h, 48 h, and 96 h, the area between the inoculated spots was imaged using confocal Raman microscopy (CRM), and the results were analyzed using principal-component analysis (PCA) in order to assess the spatiotemporal development of the distribution of signaling molecules in the region between the P. aeruginosa and E. coli cultures.

10.1128/mSphere.00426-20.1 FIG S1 (Left) Schematic of monoculture (top) and coculture (bottom) systems. (Right) Photographs of a P. aeruginosa monoculture plate (top) and a P. aeruginosa–E. coli coculture plate (bottom) at 48 h. To the right of each plate are a Raman image integrated over 1,330 to 1,380 cm^−1^ (top left), a principal-component loading plot (bottom), and a PCA heat map (top right). Download FIG S1, TIF file, 0.5 MB. Copyright © 2020 Cao et al. 2020 Cao et al. This content is distributed under the terms of the Creative Commons Attribution 4.0 International license.

**FIG 1:**
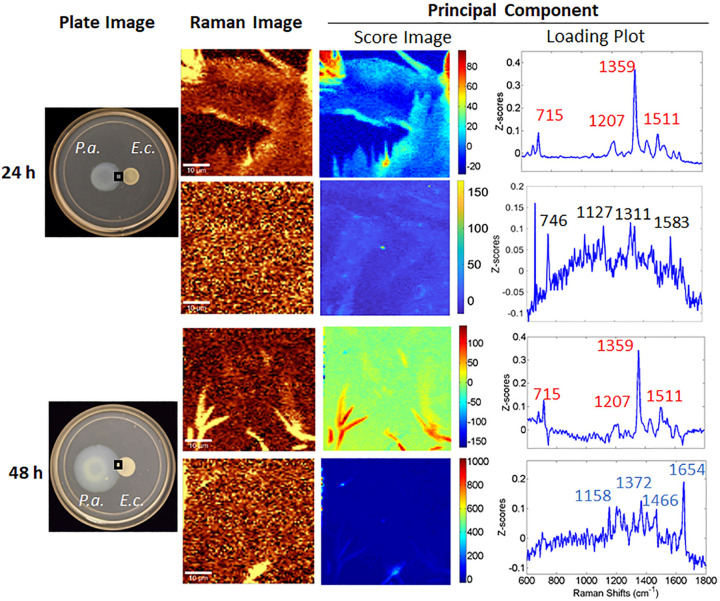
Combined CRM and PCA show that P. aeruginosa exhibits signatures of PQS by 48 h when cocultured with E. coli. Shown are images of the resultant P. aeruginosa and E. coli growth on 0.7% agar at 24 h (top) and 48 h (bottom). Raman measurements were taken in the region of the P. aeruginosa advancing edge at 24 h and at the intersection of the two strains at 48 h (shown as boxed areas on plates). One representative Raman image and one representative score image are shown for integrations over the 1,330-to-1,380-cm^−1^ (top row) and 1,630-to-1,680-cm^−1^ (bottom row) spectral windows at both 24 h and 48 h. Spectra were acquired and inspected over at least five locations within the region to validate overall consistency. Loading plots and score images of principal components were generated from principal-component analysis of the CRM microspectra acquired over the same region. HQNO and PQS features are labeled in red and blue, respectively. In the 48-h sample, PCA revealed two principal components with distinct features, which represent PQS and HQNO, respectively. Bars, 10 μm.

Previous studies have demonstrated that P. aeruginosa communities growing on semisolid surfaces tend to produce more PQS and HQNO than planktonic cultures (29). When P. aeruginosa encounters E. coli, we find that PQS appears sooner than when no E. coli is present. At 24 h, as the Raman microscopy and PCA results in Fig. 1 confirm, HQNO, with features at 715, 1,207, 1,359, and 1,511 cm^−1^, is present, but not PQS, in agreement with observations from a P. aeruginosa monoculture (shown in Fig. S2). We also observed a loading plot indicating the presence of P. aeruginosa cells with features at 746, 1,127, 1,313, and 1,583 cm^−1^. However, between 24 and 48 h, P. aeruginosa steadily expanded toward E. coli, and by 48 h, PQS features (1,158, 1,372, and 1,466 cm^−1^) appeared, much earlier than the 96-h appearance in monoculture (Fig. S2).

10.1128/mSphere.00426-20.2 FIG S2 CRM-PCA of P. aeruginosa monocultures. Optical photos, Raman images (integrated over 1,330 to 1,380 cm^−1^), Z-score spatial maps, and loading plots for the most significant principal component are given for the 24-h (a), 48-h (b), and 96-h (c) time points. HQNO (1,359 cm^−1^) is detected at ≥24 h, while PQS (1,372 cm^−1^) is detected at 96 h postinoculation. All samples were grown on FAB-glucose (12 mM) medium with 0.5% agar. Bars, 10 μm. The results shown here are representative for both the center and the edge areas. Spectra were acquired and inspected over at least five locations within each region to validate overall consistency. Download FIG S2, TIF file, 0.9 MB. Copyright © 2020 Cao et al. 2020 Cao et al. This content is distributed under the terms of the Creative Commons Attribution 4.0 International license.

### Multipoint inoculation of P. aeruginosa shows the same temporal PQS response as that in a single monoculture.

Since interaction with E. coli was shown to elicit earlier PQS production, we investigated if PQS production is stimulated by any possible competitor. We rationalized that the least competitive coculture conditions would be observed in assays of P. aeruginosa with itself. We again used a combination of CRM and PCA tools to examine the intersection area between intersecting colonies of the same P. aeruginosa strain. These P. aeruginosa self-cocultures exhibited identical surface growth phenotypes, although they were inoculated as two separate points, as shown in the plate assay images (Fig. S3, plate panel). At 48 h, Raman images and PCA confirmed the presence of HQNO in the region where two separate, expanding P. aeruginosa colonies meet (Fig. S3c), with features at positions identical to those in a single monoculture inoculation (Fig. S2). At 96 h, in addition to HQNO, PQS features were observed at 1,158, 1,372, and 1,466 cm^−1^, in agreement with the results of the single-inoculum monoculture experiment. Thus, the results of double inoculation of P. aeruginosa (Fig. S3) matched the P. aeruginosa monoculture data shown in Fig. S2, in that spectral signatures of PQS were not observed until 96 h. We contrast these monoculture results with the P. aeruginosa–E. coli coculture results of Fig. 1, in which PQS appears by 48 h. We note that the early appearance of PQS clearly correlates with the presence of E. coli, suggesting that this phenomenon is a result of intensified stress from the competition of interspecies coculturing, which is not apparent if P. aeruginosa is cocultured with itself.

10.1128/mSphere.00426-20.3 FIG S3 P. aeruginosa self-coculture plates grown for 24 h (a), 48 h (b), and 96 h (c). Black squares in the plate images indicate regions of interest. Spectra were acquired and inspected over at least five locations within each region to validate overall consistency. All assays were performed on 0.5% agar assays with FAB-glucose (12 mM). The 24-h and 48-h samples show features of HQNO; the 96-h sample shows features of HQNO and PQS. Bars, 10 μm. Download FIG S3, TIF file, 1.1 MB. Copyright © 2020 Cao et al. 2020 Cao et al. This content is distributed under the terms of the Creative Commons Attribution 4.0 International license.

### PQS production is spatially and temporally distinct from HQNO production in the presence of E. coli.

**FIG 2:**
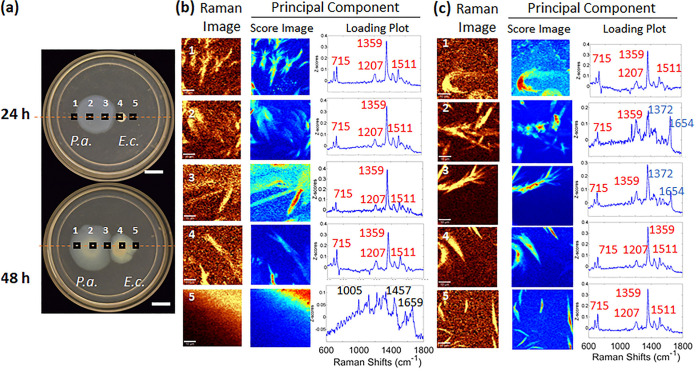
P. aeruginosa exhibits PQS signatures only in close proximity to E. coli, while HQNO signatures extend beyond the region of P. aeruginosa growth. (a) Images of P. aeruginosa–E. coli coculture plates at 24 h and 48 h (bars, 10 mm). Black squares indicate imaged areas 1 to 5 (along the dashed red lines). At least three regions of interest within each area were picked for scanning. (b) Raman images (integrated over 1,330 to 1,380 cm^−1^), Z-score spatial maps, and loading plots for the most significant principal component for 24 h as a function of position. Confocal Raman images were integrated over 1,330 to 1,380 cm^−1^ for areas 1 to 4 and over 2,800 to 3,000 cm^−1^ for area 5. Principal-component analysis was performed for all areas to generate score images and loading plots. All loading plots showed regions of Raman shifts from 600 to 1,800 cm^−1^. (c) CRM and PCA results of a 48-h coculture from area 1 to area 5 (all integrated over 1,330 to 1,380 cm^−1^), showing the locations of features from HQNO (red lettering) and PQS (blue lettering). PQS was detected within areas 2 and 3 at 48 h. Score image values range from low (dark blue) to high (red) in each plot, although the total range differs from plot to plot. All samples were grown on FAB-glucose (12 mM) medium with 0.7% agar (bars, 10 μm).

The results presented above suggest a role for PQS in mediating interactions with a foreign bacterial species. Therefore, we sought information about the detailed spatial distribution of PQS produced in cocultures. CRM imaging was performed at five sequential positions spanning a line along the P. aeruginosa–E. coli coculture plate, as shown in Fig. 2a.

At 24 h, only HQNO is detected (Fig. 2b). Unsurprisingly, HQNO signatures are apparent in areas 1 to 3, from the distal edge of the P. aeruginosa colony to its advancing edge. The optical image (Fig. 2a, top) shows that at 24 h, the cells of P. aeruginosa and E. coli are not yet in contact. However, HQNO is clearly observed in area 4, the proximal edge of E. coli facing P. aeruginosa, indicating that HQNO is able to translocate into the E. coli colony ahead of the advancing P. aeruginosa cells and is able to occupy the area of the E. coli community as an exogenous molecule. In Fig. 2b, for area 5, located at the edge of the E. coli colony distal from the P. aeruginosa cells, the dominant features detected by PCA of CRM microspectra are those of E. coli cells. The features apparent at 1,005, 1,333, 1,457, and 1,659 cm^−1^ are in excellent agreement with the Raman spectrum acquired from an E. coli monoculture acquired separately (Fig. S4a). The lack of AQ features evidenced by the Raman image over 1,330 to 1,380 cm^−1^ in area 5 (Fig. S5) instead allows E. coli cellular chemical features to dominate the PCA output. Moreover, CRM and PCA results from separate E. coli and P. aeruginosa monoculture samples (shown in Fig. S4a and S4b, respectively) clearly indicate that E. coli monoculture shows a cellular fingerprint distinct from that of P. aeruginosa and specifically shows no evidence of HQNO-related signals (cf. Fig. S2a and S4a).

10.1128/mSphere.00426-20.4 FIG S4 (a) (Left) E. coli monoculture optical image; (right) Raman spectrum exhibiting prominent peaks at 1,005, 1,457, and 1,659 cm^−1^. (b) (Left) P. aeruginosa monoculture optical image; (right) spatially resolved (left edge [1], center [2], and right edge [3]) Raman images, PCA heat maps, and PCA loading plots. The spectral windows for Raman images were taken at 1,330 to 1,380 cm^−1^ for areas 1 and 2 and at 2,800 to 3,000 cm^−1^ for area 3. All samples were grown on FAB-glucose (12 mM) medium with 0.5% agar for 48 h. Bars, 10 μm. Download FIG S4, TIF file, 0.6 MB. Copyright © 2020 Cao et al. 2020 Cao et al. This content is distributed under the terms of the Creative Commons Attribution 4.0 International license.

10.1128/mSphere.00426-20.5 FIG S5 CRM-PCA of P. aeruginosa–E. coli coculture assays at 24 h. The black square in the plate image indicates the imaged area. At least three regions of interest within the area were picked for scanning. The Raman image was integrated over 1,330 to 1,380 cm^−1^. The Z-score spatial map and the loading plot for the second significant principal component are shown. Download FIG S5, TIF file, 0.4 MB. Copyright © 2020 Cao et al. 2020 Cao et al. This content is distributed under the terms of the Creative Commons Attribution 4.0 International license.

Interestingly, the chemical profiles of these five areas shift by the 48-h time point. As the plate image (Fig. 2a, 48 h) shows, P. aeruginosa has expanded to contact the E. coli colony by this stage. Features of PQS (at 1,159, 1,372, and 1,466 cm^−1^) are readily apparent, in addition to features of HQNO, within areas 2 and 3, i.e., at the center of P. aeruginosa inoculation and at the active intersection between the two strains. Comparison of the loading plots from areas 2 and 3 indicates that PQS is most concentrated in area 3, suggesting that the stress response of P. aeruginosa to the proximity of E. coli is highly localized. Another change from the 24-h profile is that HQNO is now apparent in area 5 at 48 h (Fig. 2c). Thus, by 48 h, HQNO has spread far from areas of P. aeruginosa growth, to all areas where E. coli cells are located. We note how the distributions of HQNO and PQS were markedly different, in that PQS was not detected at these areas away from the point of P. aeruginosa–E. coli convergence (even at the distal edge of the P. aeruginosa colony). These spatial analysis experiments not only demonstrate that PQS is produced later than HQNO in these coculture assays; they also suggest that PQS is first produced preferentially at the intersection of the cocultured strains, where the competitive stress is highest, and at the P. aeruginosa colony center, where cells have accumulated to the highest density. These observations clearly indicate that P. aeruginosa has the ability to spatially regulate its PQS response (but not HQNO) within just a subset of the community in response to spatially local stimuli.

### Metabolic stress boosts PQS production and alters the PQS production pathway.

We further probed AQ responses as a function of nutrient availability. We hypothesized that the faster PQS response resulting from E. coli interaction shown above (Fig. 1 and 2) could be further intensified if nutrient availability were more limited, because competitive pressures would be more severe. For simplicity, we modified only the carbon source and performed assays with reduced glucose concentrations. While the initial experiments utilized a glucose concentration of 12 mM, here, glucose concentrations were limited to 6 mM or 3 mM, again using CRM in combination with PCA to assess the spatiotemporal attributes of AQ expression at 24 h and 48 h.

**FIG 3:**
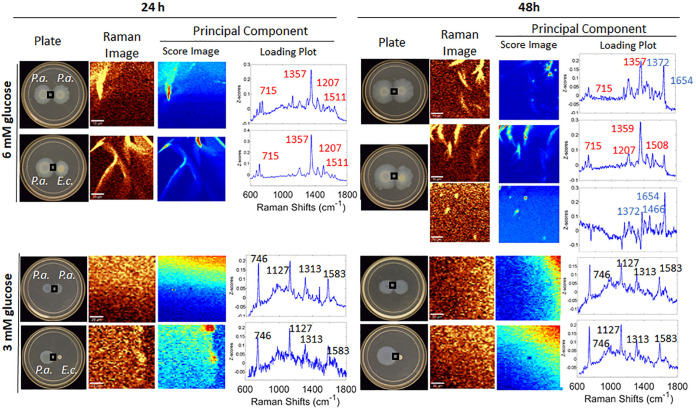
Effects of metabolic stress on PQS production with and without interspecies competition. Imaged areas are boxed on the plates, and at least three regions of interest within each area were picked for scanning. The matrices of CRM and PCA results were acquired as a function of glucose concentration (3 mM and 6 mM) and time (24 h and 48 h) for both P. aeruginosa with P. aeruginosa and P. aeruginosa with E. coli. Each panel shows (from left to right) an optical image of the plate, the Raman image, the Z-score image, and the loading plot. For 6 mM glucose samples, Raman images were integrated over the spectral window of 1,330 to 1,380 cm^−1^, except for the 48-h coculture of P. aeruginosa with E. coli, which also includes intensities integrated from 1,630 to 1,680 cm^−1^. For 3 mM glucose samples, Raman images were integrated over the range of 2,800 to 3,000 cm^−1^. For the 48-h sample in 6 mM glucose only, the first two principal-component Z-score images and loading plots are shown. Score image values range from low (dark blue) to high (red) in each plot, although the total range differs from plot to plot. All samples were grown on FAB-glucose (at 6 mM [top] or 3 mM [bottom]). Bars, 10 μm.

As described above, P. aeruginosa growing on plates containing 12 mM glucose in a single-species monoculture or self-coculture exhibits PQS at 96 h (Fig. S2 and S3), and this timing was accelerated to 48 h in E. coli competition assays (Fig. 1 and 2). Experiments with reduced levels of glucose show that these PQS responses are conditionally affected by nutrient availability. Figure 3 illustrates a matrix of CRM and PCA results acquired as a function of glucose concentration (3 mM and 6 mM), incubation pairing (P. aeruginosa–P. aeruginosa and P. aeruginosa–E. coli), and time (24 h and 48 h). Under the most extreme metabolic stress conditions, i.e., 3 mM glucose, no features characteristic of HQNO or PQS production were observed. We draw this conclusion by examining spectral windows of both 1,330 to 1,380 cm^−1^ (quinolone ring stretching region; Fig. 3) and 2,800 to 3,000 cm^−1^ (C-H stretching region; Fig. S6) based on our prior work (12, 25). The only features detected were those of P. aeruginosa cells at 746, 1,127, 1,313, and 1,583 cm^−1^, independently of time and incubation pairing. Given the adequacy of 3 mM glucose for supporting bacterial growth, which was apparent by visual inspection (Fig. 3, plate panels), we were surprised by the stark contrast in AQ production for these assays. While PQS production is already known to be cell density dependent, it was surprising that colonies of the size observed exhibited no AQ signature even after 96 h (not shown). In combination, the smaller relative expansion and the lack of an AQ signature at 3 mM glucose are clearly consistent with nutrient limitation. This result is in agreement with the findings of prior investigations of the stringent response, where the absence of (p)ppGpp, which accumulates under starvation conditions, was required for production of PQS (21, 30). This reinforces prior genetics work showing that production of any AQ, not just PQS, would require a threshold metabolic state in addition to a quorum population density (21). In comparison to setups such as biofilm flow cell assays, where extremely low nutrient concentrations are sufficient to promote QS responses, our results suggest that certain surface growth conditions may require a higher nutrient threshold to elicit an equivalent QS response. Thus, we conclude that the starvation “threshold” required to enable AQ production is a relative target that varies greatly depending on the specific growth conditions.

10.1128/mSphere.00426-20.6 FIG S6 Additional CRM-PCA analysis of P. aeruginosa assays with 3 mM glucose related to Fig. 3. Raman images were all integrated over 2,800 to 3,000 cm^−1^. Z-score spatial maps and loading plots for the second significant principal components are shown here (first principal components are shown in Fig. 3). Download FIG S6, TIF file, 0.8 MB. Copyright © 2020 Cao et al. 2020 Cao et al. This content is distributed under the terms of the Creative Commons Attribution 4.0 International license.

**FIG 4:**
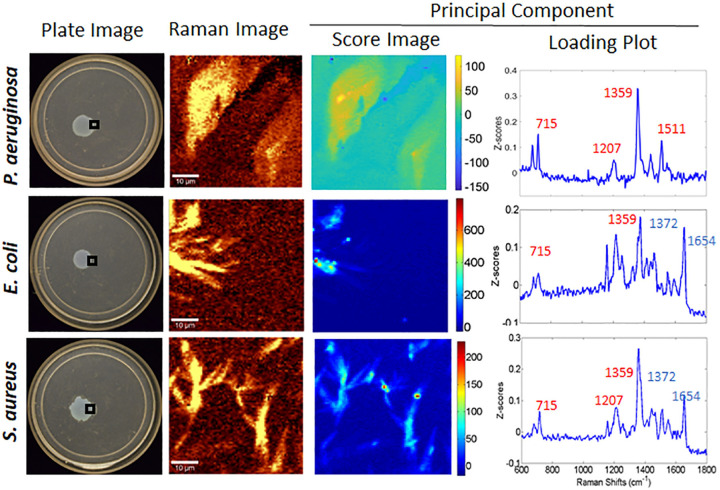
Optical images, Raman images (1,330 to 1,380 cm^−1^), and PCA of the advancing edge (boxed areas on plates) of P. aeruginosa exposed to 1 μl of a P. aeruginosa, E. coli, or S. aureus supernatant. Supernatants were spotted directly on the edge at 18 h postinoculation, and the plate was then returned to the incubator for another 6 h before being removed for testing. All samples were grown on FAB-glucose (12 mM) medium with 0.5% agar. Bars, 10 μm.

In assays with 6 mM glucose, PQS is produced in both noncompetitive and competitive modes, i.e., in both P. aeruginosa–P. aeruginosa cocultures and P. aeruginosa–E. coli cocultures, as early as 48 h (Fig. 3). While only HQNO is observed in either coculture at 24 h, PQS is observed under both coculture conditions at 48 h. These 6 mM glucose assays represent the only condition we tested under which P. aeruginosa alone exhibited a PQS response by 48 h. The collective different HQNO and PQS responses indicate that while competition and nutrient stress can both cue the PQS response, a threshold metabolic state is required for its initiation. This result was apparent for the entire colony with no spatial variation. These results also point to the utility of motility plate assays for conducting these experiments: while ample bacterial growth was exhibited in all assays examined (Fig. 1 to 4; also Fig. S2 to S4), a range of condition-specific AQ responses was observed, mediated by the different scenarios we tested.

### Interspecies supernatants induce PQS production in P. aeruginosa.

Having observed PQS production at the intersection of P. aeruginosa and E. coli growth, we asked whether this secretion process needed direct interspecies contact between P. aeruginosa and E. coli or whether a soluble factor could stimulate PQS production. We tested this by adding supernatants from 2-day E. coli planktonic cultures to P. aeruginosa growing under the same surface conditions used above with ample nutrient levels (12 mM glucose). Above, we noted production of PQS in response to the presence of E. coli within 48 h of coinoculation (Fig. 1). We hypothesized that stimulation of P. aeruginosa using a supernatant from an already-grown high-cell-density E. coli culture would stimulate PQS production earlier. To test this, an E. coli-derived supernatant was added to the growing edge of an 18-h P. aeruginosa monoculture plate, and the plate was incubated for another 6 h, thereby achieving a total of 24 h of P. aeruginosa growth time. Figure 4 shows clear spectral evidence of the presence of both HQNO and PQS at the advancing edge of P. aeruginosa, where the supernatant was pipetted, indicating that soluble factors produced by E. coli contained in the small volume (1 μl) of supernatant are sufficient to induce PQS production. Thus, direct contact between P. aeruginosa and E. coli cells is not necessary to induce P. aeruginosa PQS production. We also conclude that 6 h is sufficient to elicit a PQS response under our surface growth conditions. Subsequently, we expanded our investigation to learn if supernatants from bacteria other than E. coli would elicit a PQS response. We chose Staphylococcus aureus USA300 as a second test strain, because it coexists and competes with P. aeruginosa in many clinical settings (27), and it is also important to understand how the behaviors of the two strains are affected by each other. S. aureus supernatant was added to the P. aeruginosa plates in the same fashion as the E. coli samples (Fig. 4). In response to the S. aureus-derived supernatant, the level of PQS spiked in these samples in a manner equivalent to that observed with the addition of E. coli supernatant. To control for the possible stimulation of PQS by the addition of new nutrients, we tested the responses of P. aeruginosa to its own spent medium (Fig. 4, top) and to the growth medium alone as an uninoculated planktonic control (Fig. S7). As shown in both figures, spotting 1 μl of the P. aeruginosa supernatant or growth medium was not sufficient to stimulate PQS production. Thus, both E. coli and S. aureus supernatants elicited PQS production in P. aeruginosa during surface colonization, and we conclude that the stress imposed by other bacterial species on P. aeruginosa is readily conveyed in soluble factors produced by either species. These results for the addition of supernatants align with the findings of Horspool and Schertzer (31) showing stimulation of the production of P. aeruginosa outer membrane vesicles (OMVs) by E. coli supernatants. OMVs have been shown by the Schertzer group and others to be a primary delivery mechanism for PQS.

10.1128/mSphere.00426-20.7 FIG S7 CRM and PCA of P. aeruginosa exposed to 1-μl volumes of different media only at 24 h. (Top) LB (used to culture S. aureus); (bottom) FAB-glucose. Media were spotted directly onto the edge of a PAO1C colony at 18 h as controls for the E. coli and S. aureus supernatant experiments. P. aeruginosa was originally supported on FAB medium with 0.5% agar, supplemented with 12 mM glucose. Bars, 10 μm. Download FIG S7, TIF file, 0.5 MB. Copyright © 2020 Cao et al. 2020 Cao et al. This content is distributed under the terms of the Creative Commons Attribution 4.0 International license.

### Two-factor interaction model.

**FIG 5:**
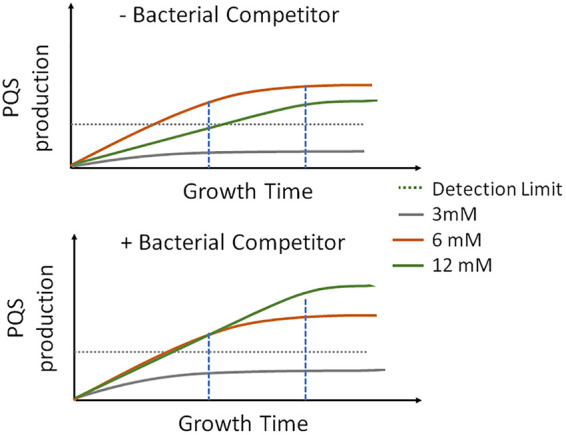
Schematic illustration of the two-factor interaction model. Shown are plots of PQS production as a function of growth time without (top) and with (bottom) a bacterial competitor (e.g., E. coli) at 6 mM or 12 mM glucose. The metabolically stressed condition (3 mM glucose) never achieves PQS production. Dashed horizontal lines indicate an effective level of PQS production.

These experiments clearly illustrate that PQS production is cued by multiple factors. Moreover, we find that PQS production is cued differently, both temporally and spatially, from HQNO production. Our group and others have previously established that surface growth greatly stimulates both HQNO and PQS production (13, 23). However, under surface motile conditions, the appearance of HQNO predictably precedes the appearance of PQS, but HQNO is not modulated equivalently to PQS (12, 32). Here, we present evidence that at least two separate factors promote PQS production during surface growth, and we present a working model to describe the onset of PQS activation (Fig. 5). PQS production, which reflects a regulatory response to the presence of an environmental challenge, is plotted as a function of growth time either without (Fig. 5, top) or with (Fig. 5, bottom) the presence of a competitor (e.g., E. coli). PQS levels are represented by three curves, corresponding to the different glucose concentrations used in the present experiments (black for 3 mM, gray for 6 mM, and red for 12 mM). In the absence of E. coli (Fig. 5, top), the production of PQS reaches an effective level (indicated by the dashed horizontal line on the plot) in less time when P. aeruginosa experiences some metabolic stress in the form of nutrient limitation (6 mM glucose) than when nutrient levels are adequate (12 mM glucose). However, when the organism is grown with more-severe nutrient limitation (3 mM glucose or less), production of PQS, and of all AQs, is repressed. In the presence of competition stress (e.g., E. coli or soluble factors from a supernatant of E. coli or S. aureus) (Fig. 5, bottom), PQS is produced earlier under both 6 mM and 12 mM nutrient conditions. The production of HQNO was essentially binary: when P. aeruginosa was growing on surfaces with sufficient nutrients to overcome stringent-response repression, HQNO was produced and detected communitywide. Alternatively, in planktonic culture and/or under starvation conditions, HQNO was absent. While this simple model does not likely capture the overall regulatory response to all possible environmental stressors, it does highlight the manner in which the two principal environmental challenges studied here interact to affect PQS production. Clearly, further research is needed to determine the differential regulation of PQS and HQNO, as well as the spatial scales on which these AQs are produced and disseminated.

## MATERIALS AND METHODS

### Bacterial strains.

Three bacterial strains were used in these experiments: Pseudomonas aeruginosa PAO1C (33), Escherichia coli K-12 (34), and Staphylococcus aureus (methicillin-resistant S. aureus [MRSA]) USA300 (35).

### Culturing and surface assay conditions.

All bacterial strains were grown planktonically overnight to an optical density at 600 nm (OD_600_) of ≈1.0 in FAB–glucose at 37°C with shaking at 240 rpm. Plates were made by adding 10 ml of sterile FAB-glucose (12 mM, 6 mM, or 3 mM glucose) solidified with 0.5% (wt/vol) Noble agar (or 0.7% [wt/vol] agar, as noted, for select experiments) to 60-mm petri dishes, a procedure similar to our previously reported method (13, 36). For monoculture plates, P. aeruginosa was spot-inoculated by pipetting 1 μl of a planktonic culture onto the center of the plate. For cocultured plates, 1 μl of each planktonic culture was simultaneously spot-inoculated at a distance of 12 mm, centered in the middle of the plate, as shown in Fig. S1. For experiments in which a supernatant was added, the supernatant was generated from 2-day planktonic cultures (E. coli was cultured in FAB-glucose, and S. aureus was cultured in LB) and isolated by centrifugation at 14,000 rpm for 2 min, followed by filtration through a 0.2-μm-pore-size filter. A 1-μl volume of supernatant was then added to those assay plates by pipetting it onto the advancing edge of the P. aeruginosa area. After inoculation, all assay plates were covered and left undisturbed until the cells were completely absorbed into the agar. Plates were then inverted and incubated at 30°C in a humidity-controlled (85% relative humidity [RH]) incubator until the desired time. Optical images of plate assay results were acquired using a Nikon D3300 camera (Nikon, Melville, NY) with an 18- to 55-mm f/3.5-5.6G VR II zoom lens.

### Raman imaging and PCA.

Raman microspectra of the standards (PQS and HQNO) were taken by averaging 10 spectra with an integration time of 0.5 s each. CRM imaging was performed as described previously (13). Briefly, Raman images were acquired by scanning over a selected area of interest on the plate, acquiring a full Raman spectrum at each image pixel using a 40× air objective (numerical aperture [NA], 0.6). Multipoint scans were carried out in the same fashion by laterally moving the sample stage to reach the desired position for each spectral acquisition. Images consisted of 80 × 80 pixels obtained at an integration time of 100 ms per spectrum. Spectra were acquired and averaged over at least five regions of interest within each numbered area. MATLAB was used to perform principal-component analysis (PCA) using previously described custom scripts (37) to extract chemical information from the data set. In addition to PCA, we reconstructed Raman images integrated over spectral window from 1,330 to 1,380 cm^−1^, indicative of differences in quinolone ring stretching for AQs, and 2,800 to 3,000 cm^−1^, indicative of C-H bond stretching for all biochemical cellular components (11, 25). Figure S2 illustrates CRM data acquisition and analysis as applied to a P. aeruginosa PAO1C monoculture. Because the coexpression of HQNO and PQS can produce PCA features with a complex line shape, these features were fit to a sum of two Voigt profiles, as shown in Fig. S8, to assess the presence of both components.

10.1128/mSphere.00426-20.8 FIG S8 Line shape analysis of the PCA feature corresponding to the quinolone ring stretch of the loading plot in Fig. 2c, location 3. The experimental data (blue circles) are fit to two Voigt line profiles centered at 1,359 cm^−1^ and 1,374 cm^−1^. Download FIG S8, TIF file, 0.2 MB. Copyright © 2020 Cao et al. 2020 Cao et al. This content is distributed under the terms of the Creative Commons Attribution 4.0 International license.
